# The landscape of schizophrenia on twitter

**DOI:** 10.1192/j.eurpsy.2021.985

**Published:** 2021-08-13

**Authors:** T. Rodrigues, N. Guimarães, J. Monteiro

**Affiliations:** 1 Psiquiatria, Centro Hospitalar Vila Nova de Gaia/Espinho, Vila Nova de Gaia, Portugal; 2 Ciências De Computadores, Faculdade de Ciências da Universidade do Porto, Porto, Portugal; 3 Psychiatric Department, Vila Nova de Gaia/Espinho Hospital Center, Vila Nova de Gaia, Portugal

**Keywords:** Twitter, Schizophrenia, Emotion Analysis

## Abstract

**Introduction:**

People with schizophrenia experience higher levels of stigma compared with other diseases. The analysis of social media content is a tool of great importance to understand the public opinion toward a particular topic.

**Objectives:**

The aim of this study is to analyse the content of social media on schizophrenia and the most prevalent sentiments towards this disorder.

**Methods:**

Tweets were retrieved using Twitter’s Application Programming Interface and the keyword “schizophrenia”. Parameters were set to allow the retrieval of recent and popular tweets on the topic and no restrictions were made in terms of geolocation. Analysis of 8 basic emotions (anger, anticipation, disgust, fear, joy, sadness, surprise, and trust) was conducted automatically using a lexicon-based approach and the NRC Word-Emotion Association Lexicon.

**Results:**

Tweets on schizophrenia were heterogeneous. The most prevalent sentiments on the topic were mainly negative, namely anger, fear, sadness and disgust. Qualitative analyses of the most retweeted posts added insight into the nature of the public dialogue on schizophrenia.

**Conclusions:**

Analyses of social media content can add value to the research on stigma toward psychiatric disorders. This tool is of growing importance in many fields and further research in mental health can help the development of public health strategies in order to decrease the stigma towards psychiatric disorders.

